# A lethal dose of insight: A case report of acetamiprid and fipronil self-poisoning

**DOI:** 10.1016/j.toxrep.2025.101968

**Published:** 2025-02-24

**Authors:** Amarthya sree Racha, Venkata Sravani Mandava, Bhanu Prakash Reddy Attunuru, Siva Kumar Reddy L

**Affiliations:** a Department of Critical Care Medicine, AIG Hospitals, Hyderabad, Telangana, India; b Department of Internal Medicine, AIG Hospitals, Hyderabad, Telangana, India

**Keywords:** Acetamiprid, Fipronil, Seizures, Hypertension, Insecticide poisoning

## Abstract

The management of insecticide poisoning is challenging due to the lack of specific antidotes and paucity of data on management. Clinical care often relies on the identification of toxic agents and the provision of supportive treatment. We present a case of a 21-year-old male who ingested 900 ml of an insecticide formulation containing acetamiprid (4 %) and fipronil (4 %) during a deliberate act of self-harm. The patient experienced severe complications, including seizures, respiratory failure, acute kidney injury, and hypertension. Despite these challenges, the patient ultimately recovered after prolonged intensive supportive care.

## Highlights

• 4 % Acetamiprid and 4 % Fipronil an affordable insecticide combination in LMICs.
• Gastrointestinal, neurological, nephrotoxic effects are the most common complications.
• Hypertension can develop after acetamiprid and fipronil poisoning.
• Fipronil raises endothelin-1, acetamiprid activates nAChRs causing vasoconstriction.
• Timely identification and a targeted approach are crucial for effective management.

## Graphical Abstract

**Figure:**
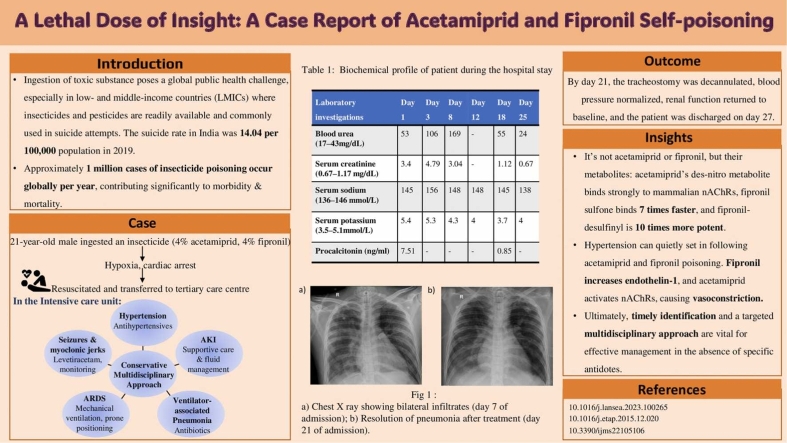

## 1 Introduction

Ingestion of toxic substances remains a significant public health issue, particularly in low- and middle-income countries where insecticides and pesticides are widely used in suicide attempts. In 2019, the suicide rate in India was 14.04 per 100,000 population [1]. Organophosphates and carbamates are well known for their high toxicity, and neonicotinoids, such as acetamiprid, have been developed as safer alternatives. However, acetamiprid, a first-generation neonicotinoid, has been associated with mortality [2], and fipronil, an N-phenylpyrazole another systemic insecticide, has been associated with severe neurotoxicity, renal dysfunction, hepatic impairment, and death [3], [4], [5]. To the best of our knowledge, there is a solitary reported case of combined poisoning with acetamiprid and fipronil, resulting in milder events and discharge after 3 days of hospitalization [6]. Here, we report a case involving significant complications (seizures, respiratory failure, and Acute Kidney Injury) requiring intensive care and prolonged hospitalization, providing new insights into other complications such as hypertension. Our case intends to raise awareness of other potential complications and underscore the importance of supportive care in preventing deterioration and promoting faster recovery.

## 2 Case presentation

A 21-year-old otherwise healthy male presented to a regional healthcare facility nine hours after deliberately ingesting 900 ml of an insecticide formulation. The patient initially presented with vomiting, hiccups, oral frothing and abdominal discomfort. The toxic agents were identified as acetamiprid (4 %) and fipronil (4 %) at the primary care center based on the container presented by the patient. Upon questioning, the patient's attendants revealed that he was staying in a hostel and was stressing about exams.

In no time, the patient rapidly developed hypoxia and experienced cardiac arrest within 24 h of ingestion. He was successfully resuscitated, intubated, and initiated on vasopressor support before being transferred to our Emergency Medicine Department (EMD).

## 3 Initial findings

At presentation (24 h after poisoning), the patient was agitated and had the following vital signs: tachycardia (160 beats per minute), tachypnea (33 breaths per minute), hyperthermia (102.4 °F), and hypotension (80/60 mmHg). Shortly after arrival, the patient experienced generalized tonic-clonic seizures (GTCS). The seizures were effectively treated with IV lorazepam (4 mg) and levetiracetam (1 g). The patient was then transferred to the intensive care unit (ICU) for further management. Owing to delayed presentation, blood samples were not sent for pesticide concentration analysis; however, the agent was confirmed based on documentation provided by the regional center.

## 4 Clinical course and management during ICU stay

### 4.1 Day 1 of admission (day 2 of poisoning)

• **Post-cardiac** a**rrest** m**anagement:** Echocardiography revealed no significant abnormalities. Hyperkalaemia (serum potassium 5.4 mmol/L) was managed conservatively with 100 ml of 25 % Dextrose, 10 units of Insulin, and IV calcium gluconate 1 g.
• **Shock, Acute Kidney Injury (AKI), and metabolic** a**cidosis:** The patient's condition deteriorated, showing signs of infection with elevated procalcitonin, along with metabolic acidosis and AKI, as indicated by creatinine and urea levels in Table 1. Intravenous fluid therapy was promptly initiated; however, the patient progressed to shock, requiring immediate administration of dual vasopressors (noradrenaline and vasopressin) and escalation of antibiotic to meropenem.

**Table 1:** Laboratory investigations of this patient during the hospital stay.

**Laboratory investigations**	**Day 1**	**Day 3**	**Day 8**	**Day 12**	**Day 18**	**Day 25**
**Renal function test**						
Blood urea (17–43 mg/dL)	**53**	**106**	**169**	**-**	**55**	24
Serum creatinine (0.67–1.17 mg/dL)	**3.4**	**4.79**	**3.04**	-	1.12	0.67
Serum sodium (136–146 mmol/L)	145	**156**	**148**	**148**	145	138
Serum potassium (3.5–5.1 mmol/L)	**5.4**	**5.3**	4.3	4	3.7	4
**Procalcitonin (ng/ml)**	**7.51**	-	-	-	0.85	-

### 4.2 Day 2 of admission

• **Neurological** f**indings:** The patient regained consciousness, and the shock gradually resolved, allowing for the cessation of vasopressor support. A brain computed tomography (CT) scan showed no abnormalities. To manage myoclonic jerks, levetiracetam was administered intravenously at a dosage of 1 g every 8 h.

### 4.3 Day 3 of admission

• Ventilatory support was maintained. Due to persistent fever, microbiological evaluation of the Bronchoalveolar lavage (BAL) fluid was performed. While awaiting culture sensitivity reports, Polymyxin B 7.5 lakh units) was administered every 12 h, followed by a loading dose of 15 lakh units.

### 4.4 Day 5 of admission

• **Hypertension:** The patient developed severe hypertension with a systolic blood pressure of 155–185 mmHg and a diastolic blood pressure of 80–100 mmHg. These symptoms may have been masked by metabolic acidosis and septic shock, which were managed with vasopressor support and antibiotic escalation to Polymyxin B along with meropenem. After a brief literature review, we suspected that hypertension was related to poisoning. Consequently, we initiated labetalol infusion (2 mg/minute for 24 h) and oral cilnidipine (10 mg every 12 h).

### 4.5 Day 6 of admission

**Fig. 1:**
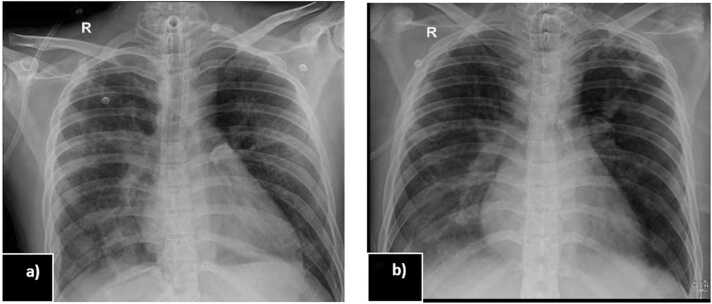
a) Chest X ray showing bilateral infiltrates (day 7 after admission); b) Resolution of pneumonia after treatment (day 21 of admission).

• **Ventilator-associated pneumonia (VAP):** Carbapenem-resistant *Acinetobacter baumannii* (CRAB) (Table 2) was isolated from the Bronchoalveolar lavage (BAL) fluid. Meropenem was discontinued, and sulbactam was added to the Polymyxin B regimen. Subsequently, the patient underwent a tracheostomy on day 7. Fig. 1a showing bilateral infiltrates during the initiation of treatment. With aggressive respiratory care and prone positioning, the patient gradually improved over the following days.

**Table 2:** Antibiotic susceptibility of the *Acinetobacter baumannii complex* isolate recovered from this patient.

**Antibiotics**	**Interpretation**	**MIC (mu/ml)**
Tigecycline	Sensitive	NA
Ceftriaxone/Sulbactam/EDTA	Sensitive	NA
Colistin	Intermediate	1
Piperacillin/Tazobactam	Resistant	≥ 128
Ceftazidime	Resistant	≥ 64
Cefoperazone/Sulbactam	Resistant	≥ 64
Cefepime	Resistant	≥ 32
Aztreonam	Resistant	≥ 64
Imipenem	Resistant	≥ 16
Meropenem	Resistant	≥ 16
Amikacin	Resistant	≥ 64
Gentamicin	Resistant	≥ 16
Ciprofloxacin	Resistant	≥ 4
Levofloxacin	Resistant	≥ 8
Minocycline	Resistant	16
Trimethoprim/Sulfamethoxazole	Resistant	≥ 320
Ampicillin/Sulbactam	Resistant	NA

Method: Conventional Aerobic Culture. Semiquantitative/standard Loop. Susceptibility by Disc Diffusion/Automated MIC.

### 4.6 Outcome

The patient's blood pressure stabilized, and both renal function and serum electrolyte levels returned to normal (Table 1). The pneumonia showed improvement (Fig. 1b), and antibiotics were discontinued after 14 days. By day 21, the tracheostomy was decannulated, and the patient was discharged on day 27 after psychiatric counselling with recommendations for follow-up psychiatric care.

## 5 Discussion

Insecticide poisoning is a significant contributor to morbidity and mortality globally, with an estimated one million cases annually. Although acetamiprid and fipronil have been developed as safer alternatives, they can still cause severe toxic effects in humans. In insects, acetamiprid is an agonist of nicotinic acetylcholine receptors (nAChRs), resulting in neuroexcitation and paralysis. Similarly, fipronil blocks gamma-aminobutyric acid (GABA_A_) and glutamate ionotropic receptors, causing comparable effects in insects [6], [7]. As discussed by Elangovan et al., the des-nitro metabolite of neonicotinoids, which has a charged nitrogen atom, exhibits significant toxicity in humans because of its high affinity for mammalian nAChRs. Similarly, fipronil sulfone, a metabolite, has a binding rate constant that is nearly seven times higher than that of fipronil. Furthermore, fipronil-desulfinyl, another metabolite, is ten times more potent in mammals [8].

In this case, the patient experienced the expected complications, which included seizures, respiratory failure, and renal impairment. These issues were conservatively managed. These complications have been reported to result in varying outcomes, ranging from complete recovery to death, in multiple cases of poisoning involving fipronil and acetamiprid, either in combination or individually [2], [4], [6].

In addition to the aforementioned complications, this patient developed severe hypertension, which was not observed in another reported case of co-poisoning with acetamiprid and fipronil [6]. [9] A study found that exposure to fipronil resulted in elevated systolic blood pressure in rats, attributed to increased concentrations of endothelin-1, a potent vasoconstrictor [9]. Hypotension was reported in a 74-year-old woman and 58-year-old man with acetamiprid poisoning [10], [11]. Hypertension has not yet been documented in humans. Although acetamiprid alone had minimal effects on blood pressure in rats, when combined with nicotine, it produced a biphasic effect characterized by initial hypotension followed by hypertension. Hypertension is caused by vasoconstriction and increased peripheral resistance [12].

In this patient, hypertension was likely due to fipronil, as well as factors such as stress and anxiety. The impact of the combination of acetamiprid and fipronil on blood pressure requires further investigation. Management strategies that target the underlying pathophysiology, such as the use of vasodilators, effectively reduced blood pressure and helped prevent additional cardiac complications.

Prolonged hospitalization and delayed recovery, compared to previously reported cases, may be attributed to several factors: delayed presentation, lack of initial decontamination, insufficient expertise in handling this toxic compound at the primary care facility, and the combined effects of acetamiprid and fipronil. This situation was further complicated by the development of VAP, which prolonged the hospitalization.

## 6 Conclusion

Despite the absence of specific antidotes, timely identification of the toxic compound and a multidisciplinary symptomatic approach specific to acetamiprid and fipronil remain the cornerstone of management.

## CRediT authorship contribution statement

**Amarthya sree Racha:** Writing – review & editing, Writing – original draft, Conceptualization. **Venkata Sravani Mandava:** Writing – review & editing, Writing – original draft, Conceptualization. **Bhanu Prakash Reddy Attunuru:** Writing – review & editing. **Siva Kumar Reddy L:** Writing – review & editing.

## Declaration of Competing Interest

The authors declare that they have no known competing financial interests or personal relationships that could have appeared to influence the work reported in this paper.

## Data Availability

This is an anonymous original case report and the references cited are already publicly available.

## References

[1] Yadav S., K K A., Cunningham S.A., Bhandari P., Mishra U.S., Aditi A., Yadav R. (2023). Changing pattern of suicide deaths in India. Lancet Reg. Health - Southeast Asia.

[2] Gulen M., Satar S., Ince C. (2022). A fatal case of acetamiprid poisoning with turquoise urine. J. Forensic Leg. Med..

[3] Gutta S., Prasad J., Gunasekaran K., Iyadurai R. (2019). Hepatotoxicity and neurotoxicity of Fipronil poisoning in human: a case report. J. Fam. Med. Prim. Care.

[4] Mohamed F., Senarathna L., Percy A., Abeyewardene M., Eaglesham G., Cheng R., Azher S., Hittarage A., Dissanayake W., Sheriff M.H.R., Davies W., Buckley N.A., Eddleston M. (2004). Acute human self-poisoning with the *N* -phenylpyrazole insecticide fipronil—a GABA_A_-gated chloride channel blocker. J. Toxicol.: Clin. Toxicol..

[5] Yadla M., Sailaja S., Ahmed N., Uppin M., Arlappa N. (2017). An unusual case of insecticide poisoning presenting as acute kidney injury. Saudi J. Kidney Dis. Transplant..

[6] Elangovan A., Jayaprakash R., Nagaraju P. (2022). Fipronil and acetamiprid poisoning: new perils. Indian J. Crit. Care Med..

[7] Karunarathne A., Bhalla A., Sethi A., Perera U., Eddleston M. (2021). Importance of pesticides for lethal poisoning in India during 1999–2018: a systematic review. BMC Public Health.

[8] Gupta R.C., Milatovic D. (2014). Biomarkers in Toxicology.

[9] Chaguri J.L., Godinho A.F., Horta D.F., Gonçalves-Rizzi V.H., Possomato-Vieira J.S., Nascimento R.A., Dias-Junior C.A. (2016). Exposure to fipronil elevates systolic blood pressure and disturbs related biomarkers in plasma of rats. Environ. Toxicol. Pharmacol..

[10] Imamura T., Yanagawa Y., Nishikawa K., Matsumoto N., Sakamoto T. (2010). Two cases of acute poisoning with acetamiprid in humans. Clin. Toxicol..

[11] Pirasath S., Senthuran R., Athirayan C., Gevakaran M., Guruparan M., Gnanathasan A. (2021). Acute poisoning with acetamiprid: a case report. J. Med. Case Rep..

[12] Park J., Taly A., Bourreau J., De Nardi F., Legendre C., Henrion D., Guérineau N.C., Legros C., Mattei C., Tricoire-Leignel H. (2021). Partial agonist activity of neonicotinoids on rat nicotinic receptors: consequences over epinephrine secretion and in vivo blood pressure. Int. J. Mol. Sci..

